# Trends in the Prevalence of Autism Spectrum Disorder, Cerebral Palsy, Hearing Loss, Intellectual Disability, and Vision Impairment, Metropolitan Atlanta, 1991–2010

**DOI:** 10.1371/journal.pone.0124120

**Published:** 2015-04-29

**Authors:** Kim Van Naarden Braun, Deborah Christensen, Nancy Doernberg, Laura Schieve, Catherine Rice, Lisa Wiggins, Diana Schendel, Marshalyn Yeargin-Allsopp

**Affiliations:** Developmental Disabilities Branch, National Center on Birth Defects and Developmental Disabilities, Centers for Disease Control and Prevention, Atlanta, Georgia, United States of America; University of Tuebingen Medical School, GERMANY

## Abstract

This study examined the prevalence and characteristics of autism spectrum disorder (ASD), cerebral palsy (CP), hearing loss (HL), intellectual disability (ID), and vision impairment (VI) over a 15–20 year time period, with specific focus on concurrent changes in ASD and ID prevalence. We used data from a population-based developmental disabilities surveillance program for 8-year-olds in metropolitan Atlanta. From 1991–2010, prevalence estimates of ID and HL were stable with slight increases in VI prevalence. CP prevalence was constant from 1993–2010. The average annual increase in ASD prevalence was 9.3% per year from 1996–2010, with a 269% increase from 4.2 per 1,000 in 1996 to 15.5 per 1,000 in 2010. From 2000–2010, the prevalence of ID without ASD was stable; during the same time, the prevalence of ASD with and without co-occurring ID increased by an average of 6.6% and 9.6% per year, respectively. ASD prevalence increases were found among both males and females, and among nearly all racial/ethnic subgroups and levels of intellectual ability. Average annual prevalence estimates from 1991–2010 underscore the significant community resources needed to provide early intervention and ongoing supports for children with ID (13.0 per 1,000), CP, (3.5 per 1,000), HL (1.4 per 1,000) and VI (1.3 in 1,000), with a growing urgency for children with ASD.

## Introduction

Developmental disabilities (DDs) are a group of chronic conditions attributable to a physical or mental impairment which may result in functional limitations often requiring life-long coordinated, interdisciplinary services and supports [1–2]. Measuring the prevalence of DDs and evaluating trends are necessary to plan for the educational, medical, and community resources essential to support individuals with DDs and their families [3]. Trends in the prevalence of DDs and their co-occurrence may be related to changes in underlying risk factors, identification practices in the community, public health policies, use of preventive measures, and/or differences in study methodologies [4–5]. In recent decades, improvements in maternal and child health care, national screening programs, and increased community awareness of behavioral disorders may have affected the magnitude of prevalence of various DDs.

Between 1997 and 2008, US survey data, based on parental report, indicated little or no change in the prevalence of intellectual disability (ID), cerebral palsy (CP), or blindness, a decrease of 31% for moderate to profound hearing loss (HL), and a 289% increase in autism [6]. Similar increases in ASD prevalence have been reported from data using special education autism eligibility counts or systems using clinical diagnoses of developmental conditions [7–9]. The Metropolitan Atlanta Developmental Disabilities Surveillance Program (MADDSP) extends beyond methods using a single source of information by conducting systematic review of developmental evaluations at multiple education and health sources. Results from MADDSP and from the other US population-based sites participating in the Autism and Developmental Disabilities Monitoring (ADDM) Network indicate significant increases in ASD prevalence over the past decade. The most recent ADDM ASD prevalence estimate of 1 in 68 or 14.7 per 1,000 8-year-old children with ASD in 2010 was over double the Network’s 2002 estimate of 1 in 150 or 6.6 per 1,000 [5].

Both improved identification and increased risk have been posited as contributors to the reported increase in ASD prevalence [4]. One focus of this discussion has been changes in diagnostic practices for children with co-occurring ASD and ID. As such, children with both ID and ASD who would formerly have been classified as having ID only are now classified with ASD as well which may serve to facilitate access to appropriate services and supports for individuals who have ASD in addition to ID [10]. Concurrent assessment of trends in ASD and ID has been challenged by bias and/or underestimation in ascertainment of ASD and ID in a common administrative dataset or national survey [10, 11]. MADDSP includes a consistent and thorough assessment of IQ scores over time and thus, its ID prevalence estimates are more complete than those based on survey or administrative or diagnostic count methods.

Using MADDSP data, this study aims to examine trends in the prevalence and characteristics of ID, CP, HL, vision impairment (VI), and ASD over a 15–20 year time period, with specific focus on concurrent changes in the prevalence of ID and ASD stratified by demographic characteristics and intellectual ability.

## Materials and Methods

### Case Ascertainment

From 1991–1994, MADDSP monitored CP, HL, ID, and VI among 3- to 10-year-old children, with the addition of ASD in 1996. Results based on 3- to 10-year-olds indicated that the most complete ascertainment for these DDs was at age 8 years. Therefore, since 1996 for CP, HL, ID and VI and 2000 for ASD, MADDSP has monitored 8-year-olds. Surveillance is conducted every other calendar year to provide cross-sectional prevalence estimates.

In addition to age/birth year criteria, MADDSP eligibility includes a parent(s) or legal guardian(s) residing within a five-county (Clayton, Cobb, DeKalb, Fulton, and Gwinnett) metropolitan Atlanta area at any time during the applicable surveillance year. The MADDSP methodology includes active review of administrative records from multiple education and health sources. For each surveillance year, MADDSP identifies records from education sources based on special education eligibility in nine school districts serving the five counties and from private and public health sources serving the area based on a wide range of ICD-9 billing codes related to DDs.

A two-stage process is used for case ascertainment. In the first stage, trained staff screen records to confirm age and residency requirements and then search for specific indicators of one or more of the five DDs. These indicators include qualifying test results for ID, HL, or VI; behavioral or diagnostic descriptions indicating possible ASD; and physical or diagnostic findings consistent with CP. Detailed information is collected from records of eligible children with an indication of a possible DD including demographic characteristics, special education classification, qualifying test results, diagnostic information, and verbatim behavioral descriptions (ASD) or physical findings (CP) from developmental assessments. For each child with data collected from multiple sources, the information is consolidated into a single composite record.

In the second stage, ASD and CP composite records are systematically reviewed by one or more clinical research reviewers to determine final case status. ASD case status is confirmed by applying a standardized coding scheme based on the Diagnostic and Statistical Manual of Mental Disorders, Fourth Edition, Text Revision [12]. CP case status is confirmed by the presence of a documented diagnosis of CP or physical findings consistent with CP at or after age 2 years [13–14]. ID is defined as an intelligence quotient (IQ) of ≤70 on the most recently administered test of intellectual ability [15]. HL is defined as an unaided, measured bilateral pure tone hearing loss at frequencies of 500, 1,000, and 2,000 hertz averaging ≥40 decibels (dB), unaided in the better ear [15]. VI is defined as a visual acuity of 20/70 or worse in the better eye with correction [15]. Ongoing quality checks are conducted to ensure reliability across clinical research reviewers and field abstractors. The details of the MADDSP methodology have been previously described [15–16]. MADDSP functions as a public health authority in accordance with its data source agreements and meets applicable privacy/confidentiality requirements under 45 CFR 46 [17]. MADDSP is deemed public health practice by the Centers for Disease Control and Prevention’s Institutional Review Board and all data are de-identified prior to analysis.

### Statistical Analyses

Period prevalence estimates per 1,000 8-year-old children were calculated for ID, CP, HL, and VI from 1991–2010 and for ASD for 1996–2010. Numerators include children who met the case definition for one or more of the five DDs for each surveillance year. To stabilize the estimates for surveillance years with small case counts, (<5), for trend analyses the relevant numerator and denominator data were combined with the adjacent surveillance year’s numerator and denominator, respectively, to calculate a weighted average period prevalence across the two surveillance years.

Denominators used for calculating prevalence estimates were obtained from the National Center for Health Statistics (NCHS) bridged-race intercensal population estimates for 1991–1996 and 2002–2008 and the NCHS bridged-race decennial population estimates for 2000 and 2010 [18–20] (S1 Appendix). Although previous prevalence reports for 1996 (ASD) and 2002–2008 (ASD and CP) used bridged-race postcensal population estimates, intercensal denominator data have since become available and were used in the current analyses as they are considered the preferred source of denominator data [21–22] (S2 Appendix). Intercensal estimates were consistently lower than the postcensal projections from 2002–2008 resulting in higher prevalence estimates than those previously reported when postcensal denominator data were used [23–25]. Comparison of all analyses based on postcensal vs. intercensal denominators showed differences in the absolute magnitude of prevalence estimates most notably in surveillance years at the end of the decade, specifically pronounced among non-Hispanic white (NHW) children. However, the patterns of all trend analyses were similar. It is important to emphasize that the numerator case counts are unchanged and the changes in previously and currently reported prevalence estimates are solely attributable to differences in estimation of intercensal and postcensal population denominator data.

Race/ethnicity categories for numerators and denominators were defined as non-Hispanic white (NHW), non-Hispanic black (NHB), non-Hispanic American Indian/Alaska Native (NHAI/AN), non-Hispanic Asian/Pacific Islander (NHA/PI), or Hispanic. Race/ethnicity information for children in the numerator was obtained from source records and supplemented with birth certificate data when not available. NCHS bridged-race counts for these categories were calculated by county, single year of age, race, ethnic origin, and sex [18–20].

Negative binominal and Poisson regression (with a parameter to correct for overdispersion based on exploratory analyses) were used to model assumed linear trends in the observed prevalence estimates. Model selection was determined using chi-square Goodness of Fit test results (p>0.05) and comparisons of Akaike Information Criterion (AIC) and Bayesian Information Criterion (BIC) (higher estimates indicating poorer fit) for each trend. To evaluate the relationship between ASD and ID prevalence over time we compared two time periods: 1996 to 2010 and 2000 to 2010. Two time periods were used because of the anomalously higher ID prevalence estimate in 1996 compared with 9 of the other ID prevalence estimates from 1991–2010.

Average annual period prevalence was estimated as the sum of numerators across surveillance years divided by the sum of denominators across all applicable surveillance years. Average annual percent change, that is the average yearly rate of change, with 95% confidence intervals was estimated using linear temporal models. Absolute percent changes between the first and last surveillance years were also estimated for select trends to provide comparability with other reports. Co-occurrence among the five DDs was evaluated two ways: 1) proportion of children with a specific DD who also met the MADDSP surveillance case definition for one or more of the other DDs among by all children who met the case definition for a specified DD, and 2) prevalence of co-occurring DDs as the number of children with 2 specified DDs divided by the total number of 8-year-olds in a given surveillance year.

Stratum-specific prevalence estimates for all DDs are reported by sex, NHW and NHB race/ethnicity and level of severity or clinical subtype. For point estimate comparisons, a Poisson distribution was used to test the difference of two proportions with significance deemed at p<0.05. Due to small case sample sizes, Hispanic prevalence estimates are only reported for ASD, ID and CP, and NHA/PI estimates only for children with ASD and ID. ID severity was categorized as mild (IQ 50–70) or moderate to profound (IQ <50). Children reported to have ID but without a specific IQ score were categorized as ID-NOS. HL severity was categorized as moderate (40–64 dB), severe (65–84 dB), or profound (85–121 dB). VI severity was categorized as low vision (better than 20/200) or blindness (20/200 or worse) [12]. For ASD, level of intellectual ability was used as a proxy for severity in functioning with the above severity categories for ID and the following categories for children with IQ in the non-ID range: average to above average (IQ>85) and borderline (IQ 71–85) intellectual ability.

## Results

**Fig 1:**
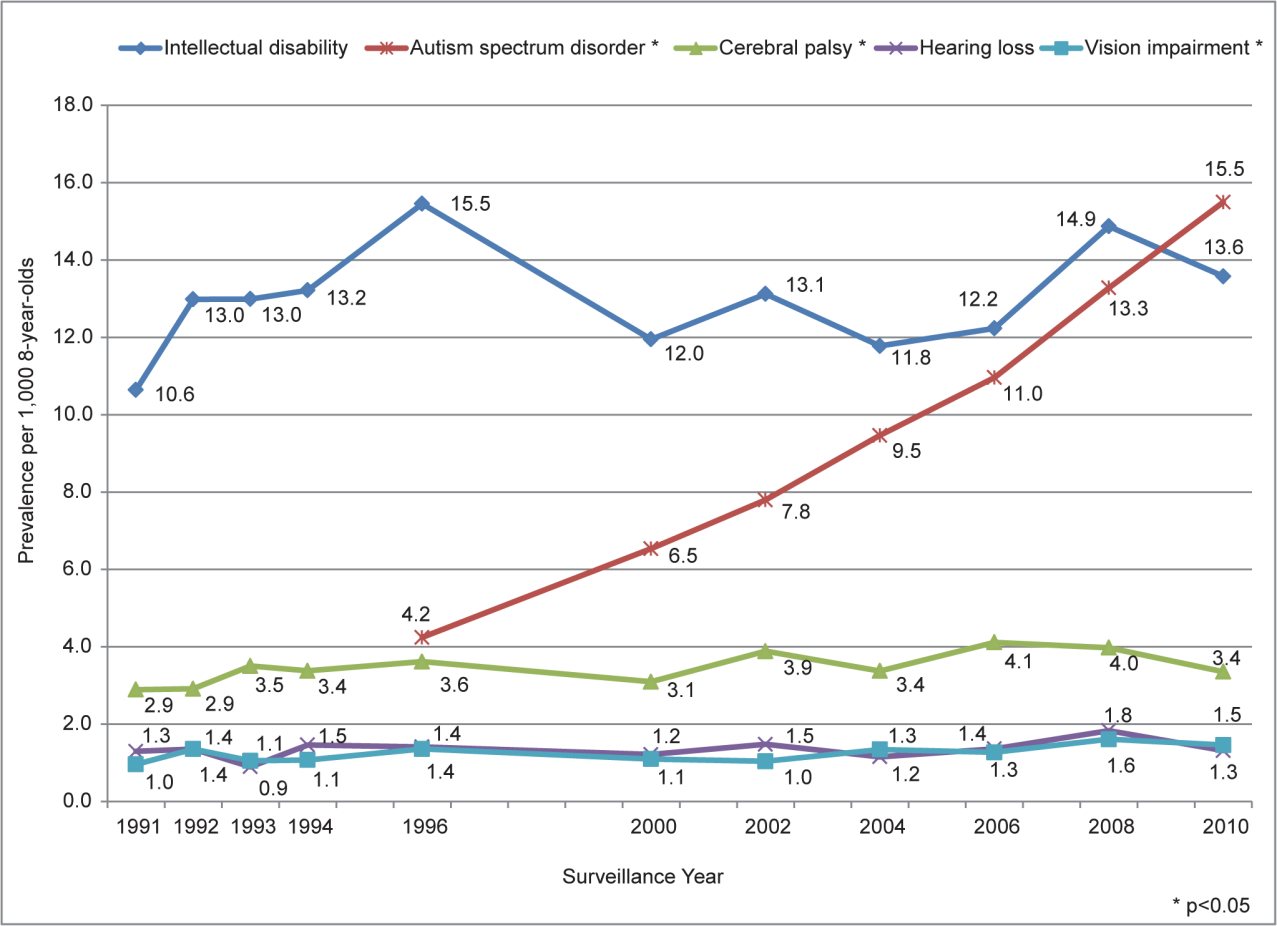
Prevalence of Five Developmental Disabilities, Metropolitan Atlanta Developmental Disabilities Surveillance Program, 1991–2010.

The population of 8-year-old children in metropolitan Atlanta grew approximately 61% from 1991 through 2010, with substantial increases for Hispanic (934%) and NHB (82%) children and a decrease for NHW (-6%) children. Overall, prevalence estimates for ID, CP, HL, and VI were relatively stable from 1991 through 2010 (Fig 1), whereas the prevalence of ASD markedly increased from 1996 through 2010.

### Intellectual disability

#### Prevalence and Demographics

The prevalence of ID was stable during the study period; 10.6 per 1,000 in 1991 and at 13.6 per 1,000 in 2010 (Table 1). Although relative spikes in prevalence were seen in 1996 (15.5 per 1,000) and 2008 (14.9 per 1,000), the remaining nine time points were similar, with prevalence estimates of approximately 11 to 13 per 1,000. Across the entire period from 1991–2010, average ID prevalence was 13.0 per 1,000 or 1 in 77. ID prevalence was consistently and significantly higher among males than females (prevalence ratios (PR), 1.3:1 to 2.3:1) and among NHB than NHW children (PR, 1.7:1 to 2.6:1). ID prevalence among Hispanic children increased from 9.4 per 1,000 in 1991–1992 to 11.7 per 1,000 in 2010, with a significant average annual increase of 2.0% (95% CI 0.2, 3.9).

**Table 1:** Trends in prevalence, demographic characteristics, and severity of children with intellectual disability, 1991–2010*.

	1991		1992		1993		1994		1996		2000		2002		2004		2006		2008		2010		1991–2010		Average annual percent change 1991–2010
	%	Prev	%	Prev	%	Prev	%	Prev	%	Prev	%	Prev	%	Prev	%	Prev	%	Prev	%	Prev	%	Prev	%	Prev	
Total	n = 320	10.6	n = 392	13.0	n = 419	13.0	n = 442	13.2	n = 568	15.5	n = 522	12.0	n = 567	13.1	n = 499	11.8	n = 529	12.2	n = 673	14.9	N = 659	13.6	N = 5590	13.0	0.35 (-0.52, 1.24)
**Demogrpahics**																									
White non-Hispanic	41.9	7.9	33.9	7.9	37.2	8.9	34.6	8.7	31.2	9.8	24.1	6.8	26.5	8.8	22.0	6.8	24.0	8.4	19.5	8.6	23.2	9.6	27.7	8.4	0.12 (-0.90, 1.16)
Black non-Hispanic	55.9	15.7	62.2	21.1	59.9	20.0	60.2	19.8	62.0	22.7	62.3	16.5	60.0	17.0	60.9	15.8	59.4	15.6	56.9	18.7	53.0	16.8	59.2	17.9	-0.95 (-1.92, 0.03)
Hispanic	nr	nr	2.3	9.4	nr	nr	3.6	12.3	3.5	15.6	6.9	10.5	7.9	10.9	10.2	11.3	12.3	12.2	14.1	14.4	15.3	11.7	8.0	11.7	**2.00 (0.15, 3.87)**
Asian non-Hispanic	nr	nr	1.5	6.9	1.7	7.7	1.6	7.0	2.6	12.5	3.3	9.3	3.2	9.4	3.4	7.2	2.5	4.9	5.6	14.1	5.0	10.4	3.1	8.9	2.20 (-1.22, 5.76)
Male	57.8	12.1	58.2	14.9	62.1	15.9	60.2	15.7	62.3	19.1	59.0	14.0	64.4	16.5	63.3	14.8	65.0	15.6	71.0	20.5	69.0	18.6	63.7	16.3	**1.19 (1.0, 2.30)**
Female	42.2	9.1	41.8	11.0	37.9	10.0	39.8	10.7	37.7	11.8	41.0	9.9	35.6	9.6	36.7	8.7	35.0	8.7	29.0	8.9	31.0	8.5	36.3	9.6	**-1.18 (-1.84, -0.51)**
**Level of severity**																									* *
Mild (IQ 50–70)	57.8	6.2	68.6	8.9	66.1	8.6	69.5	9.2	66.2	10.2	63.2	7.6	60.5	7.9	59.1	7.0	60.5	7.4	60.0	8.9	69.3	9.4	63.7	8.3	0.10 (-1.14, 1.37)
Moderate to Profound (IQ <50)	40.9	4.4	30.9	4.0	32.7	4.2	29.0	3.8	28.2	4.4	32.8	3.9	35.3	4.6	31.3	3.7	32.9	4.0	35.4	5.3	27.9	3.8	32.2	4.2	0.18 (-0.80, 1.17)
ID NOS	nr	nr	nr	nr	1.2	0.2	1.6	0.2	5.6	0.9	4.0	0.5	4.2	0.6	9.6	1.1	6.6	0.8	4.6	0.7	2.7	0.4	4.1	0.5	**7.21 (0.35, 14.5)**

*Proportions and prevalence estimates with numerators <5 children are suppressed due to small sample sizes. Trend analyses reflect weighted prevalence for surveillance years with n<5 children and the subsequent surveillance year (e.g., trend for Hispanic children with ID combined 1991–1992 numerators and denominators for the first time period).

#### Severity and Co-occurring DDs

For all surveillance years combined, the majority (63.7%) of children with ID was classified with mild ID (IQ 50–70) (Table 1). For both mild and moderate to profound ID, prevalence estimates were stable with small, non-significant average annual prevalence change estimates of 0.10% (-1.1, 1.4) per 1,000 and -0.18% (-0.8, 1.2) per 1,000 8-year-olds, respectively. Although there was a significant average annual percent increase in the prevalence of children with ID-NOS, few children were classified as such.

**Fig 2:**
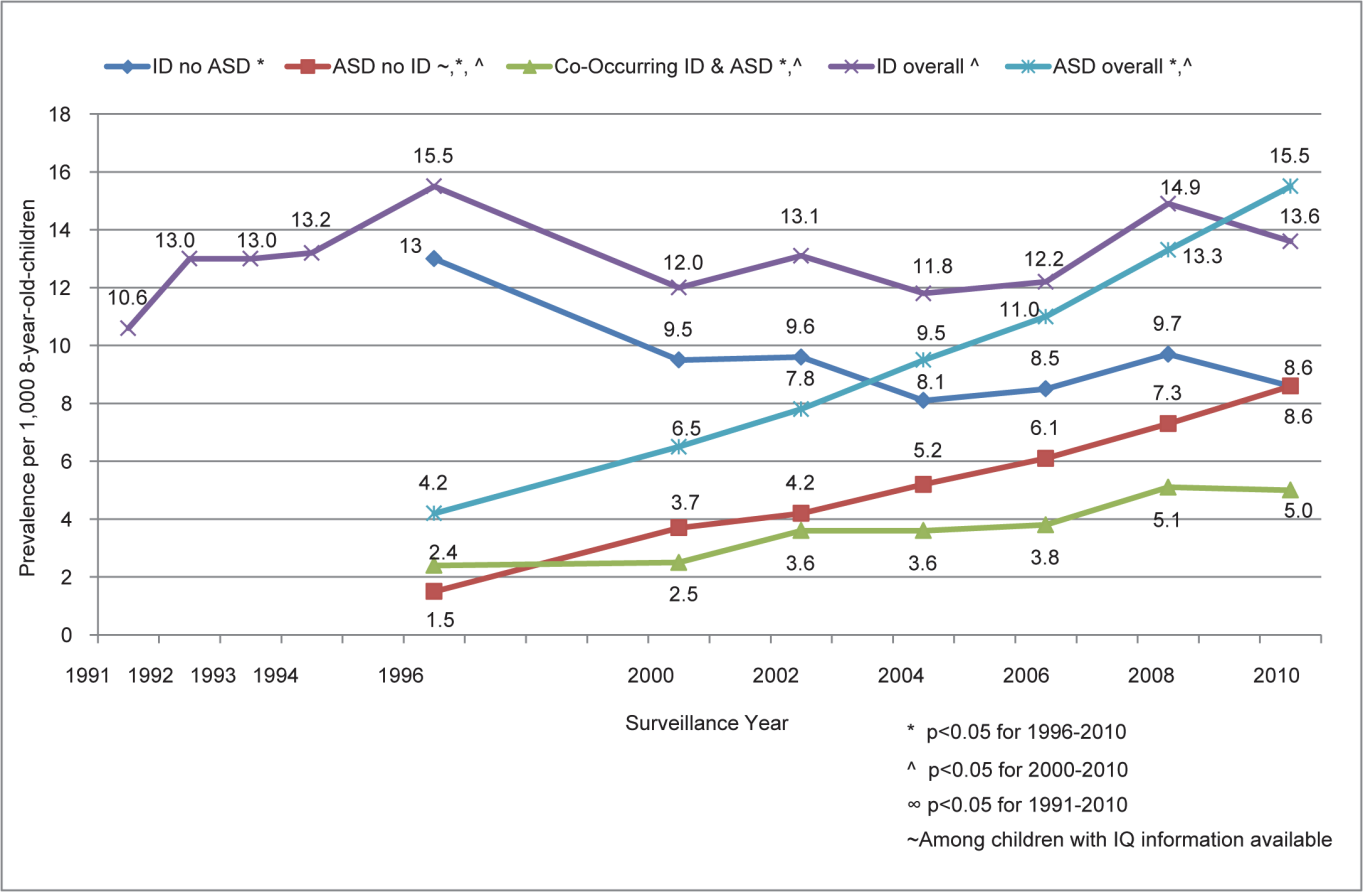
Prevalence of Co-occurring Intellectual Ability and Autism Spectrum Disorder, 1991–2010.

Among children with ID across all surveillance years combined, 28% had co-occurring ASD, followed by CP (13%), VI (5%), and/or HL (3%). Whereas no trends were found in the prevalence of co-occurring ID and CP and/or VI from 1991–2010, a significant increase in the prevalence of co-occurring ID and ASD was found from 1996–2010 (Fig 2) and ID and HL from 1991–2010. The prevalence increase among children with co-occurring ID and HL ranged from 0.02 to 0.03 per 1,000; average annual increase of 2.4% (0.1, 4.8), largely attributable to higher estimates in 2006 (0.05) and 2008 (0.04).

### Autism spectrum disorder

#### Prevalence, Demographics, and Intellectual Ability

ASD prevalence increased significantly from 4.2 per 1,000 in 1996 to 15.5 per 1,000 in 2010 (Fig 1). This represents an average annual change of 9.3% and an absolute change of 269% (Table 2). Prevalence was significantly higher among males than among females, ranging from 3.7:1 in 1996 to 4.6:1 in 2010. By 2010, approximately 1 in 40 males had ASD compared with 1 in 182 females. Prevalence of ASD was higher among NHW than NHB and Hispanic children for all years. Significant increases in ASD prevalence during 1996 to 2010 were found for males, females, NHB, NHW, and NHA/PI children, and during 1996–2000 to 2010 for Hispanic children (Table 2). The highest ASD prevalence estimate for any subgroup was among NHW males in 2010 (30.6 per 1,000 or 1 in 33).

**Table 2:** Trends in prevalence, demographic characteristics, and level of intellectual ability among children with autism spectrum disorders, 1991–2010*.

	1996		2000		2002		2004		2006		2008		2010		Average annual percent change 1996–2010
	%	Prev	%	Prev	%	Prev	%	Prev	%	Prev	%	Prev	%	Prev	
**Total**	n = 156	4.2	n = 285	6.5	n = 337	7.8	n = 401	9.5	n = 474	11.0	n = 601	13.3	N = 752	15.5	**9.34 (8.85, 9.82)**
**Demographics**															** **
White non-Hispanic	53.2	4.6	50.2	7.7	47.8	9.4	44.4	11.0	45.4	14.2	36.8	14.4	38.2	18.1	**9.45 (8.0, 10.92)**
Black non-Hispanic	39.7	4.0	35.8	5.2	39.8	6.7	37.9	7.9	39.7	9.4	41.1	12.0	38.7	14.0	**9.91 (9.19, 10.64)**
Hispanic	nr	nr	4.2	3.5	5.9	4.9	8.2	7.3	6.1	5.4	9.3	8.5	12.2	10.7	**11.67 (6.70, 16.86)**
Asian non-Hispanic	3.8	5.0	3.2	4.9	3.0	5.2	5.0	8.5	4.4	7.9	7.8	17.4	5.2	12.3	**10.35 (4.54, 16.50)**
Male	79.5	6.7	84.6	11.0	83.1	12.7	80.3	15.0	83.8	18.0	84.7	21.9	82.4	25.3	**9.45 (8.79, 10.12)**
Female	20.5	1.8	15.4	2.0	16.9	2.7	19.7	3.8	16.2	3.6	15.3	4.2	17.6	5.5	**8.68 (6.74, 10.65)**
**Level of Intellectual Ability**															
Total with Cognitive Data*	94.2		94.0		93.2		93.5		91.6		93.3		87.9		
Average to Above Average (IQ>85)	22.4	0.9	38.8	2.4	32.8	2.4	35.5	3.1	40.1	4.0	36.2	4.5	40.8	5.6	**11.18 (8.73, 13.69)**
Borderline (IQ 71–85)	16.3	0.7	21.3	1.3	18.2	1.3	23.5	2.1	22.4	2.2	22.5	2.8	22.5	3.1	**10.54 (8.34, 12.79)**
Mild (IQ 50–70)	23.1	0.9	15.7	1.0	20.4	1.5	17.9	1.6	19.4	1.9	20.7	2.6	21.6	2.9	**9.88 (8.07, 11.73)**
Moderate-Profound (IQ<50)	35.4	1.4	20.9	1.3	25.8	1.9	16.5	1.5	15.2	1.5	18.9	2.3	14.1	1.9	**3.14 (0.54, 5.81)**
Cognitive Impairment, NOS	nr	nr	3.4	0.2	2.9	0.2	6.7	0.6	3.0	0.3	1.8	0.2	0.9	0.1	-2.62 (-15.43, 12.15)
**Demographics and Level of Intellectual Ability**															
**White non-Hispanic Males**	n = 68	7.4	n = 122	13.0	n = 131	14.9	n = 145	17.8	n = 177	23.0	n = 182	23.1	N = 246	30.6	**9.63 (8.10, 11.19)**
Average to Above Average (IQ>85)	34.4	2.3	56.0	6.9	50.4	6.8	56.0	9.2	52.1	11.2	57.6	12.6	55.9	15.3	**11.28 (8.12, 14.54)**
Borderline (IQ 71–85)	21.3	1.4	19.0	2.4	18.5	2.5	18.7	3.1	20.0	4.3	19.8	4.3	19.1	5.2	**9.39 (7.82, 10.97)**
Cognitive Impairment (IQ<70)	44.3	2.9	25.0	3.1	31.1	4.2	25.4	4.2	27.9	6.0	22.7	5.0	25.0	6.8	**6.40 (4.08, 8.77)**
**Black non-Hispanic Males**	n = 50	6.4	n = 84	8.5	n = 114	11.2	n = 122	12.5	n = 159	15.5	n = 213	20.1	N = 227	21.7	**9.60 (8.49, 10.72)**
Average to Above Average (IQ>85)	12.2	0.8	22.5	1.8	10.2	1.1	19.0	2.3	22.1	3.1	20.6	3.9	29.6	5.5	**15.08 (10.71, 19.62)**
Borderline (IQ 71–85)	14.3	0.9	22.5	1.8	19.4	2.1	20.7	2.5	24.8	3.5	24.6	4.6	24.0	4.5	**11.56 (8.95, 14.24)**
Cognitive Impairment (IQ<70)	73.5	4.6	55.0	4.4	70.4	7.5	60.3	7.2	53.1	7.5	54.8	10.3	46.4	8.7	**5.67 (2.69, 8.73)**
**White non-Hispanic Females**	n = 15	1.7	n = 21	2.3	n = 30	3.6	n = 33	4.1	n = 38	5.1	n = 39	5.2	N = 41	5.2	**8.48 (5.64, 11.39)**
No Cognitive Impairment	33.3	0.6	52.6	1.1	60.7	2.0	72.7	3.0	70.6	3.2	66.7	3.2	64.7	2.8	**10.36 (4.26, 16.81)**
Cognitive Impairment (IQ<70)	66.7	1.1	47.4	1.0	39.3	1.3	27.3	1.1	29.4	1.3	33.3	1.6	35.3	1.5	**3.07 (1.06, 5.12)**
**Black non-Hispanic Females**	n = 12	1.5	n = 18	1.8	n = 20	2.0	n = 30	3.2	n = 29	2.9	n = 34	3.4	N = 64	6.2	**11.03 (6.97, 15.25)**
No Cognitive Impairment	nr	nr	27.8	0.5	nr	nr	31.0	1.0	53.6	1.5	48.5	1.6	54.2	3.1	**24.05 (17.3, 31.17)**
Cognitive Impairment (IQ<70)	81.8	1.2	72.2	1.3	77.8	1.4	69.0	2.1	46.4	1.3	51.5	1.7	45.8	2.6	**5.20 (1.17, 9.38)**

*Proportions and prevalence estimates with numerators <5 children are suppressed due to small sample sizes. Trend analyses reflect weighted prevalence for surveillance years with n<5 children and the subsequent surveillance year (e.g., trend for Hispanic children with ASD combined 1996–2000 numerators and denominators for the first time period).

Among children with ASD who had IQ information, the proportion with intellectual disability (IQ≤70) decreased markedly from 1996 to 2000. In 1996, 61% of children with ASD had ID compared with approximately 40% in 2000 and 2004–2010 (Table 2); we observed no meaningful changes in prevalence from 2000 through 2010 (p = 0.13). Among children with ASD, the proportion with co-occurring HL (1%), VI (1%), and/or CP (3%) was minimal with stable prevalence from 1996–2010.

Significant increases in ASD prevalence were found at all levels of intellectual ability with the exception of ID-NOS for which the sample sizes were small. The average annual increases among children with ASD with average to above average or borderline intellectual ability as well as that for children with ASD and mild ID were approximately 9.9–11.2% per year. The increase for children with ASD and moderate to profound ID was smaller (3.1%) (Table 2).

#### Comparison of prevalence trends for ASD and ID

ASD and ID overall: ASD prevalence, both with and without ID, increased significantly for both time periods with average annual increases from 2000 to 2010 of 6.6% for ASD with ID and 9.6% for ASD without ID (Fig 2). ID prevalence without co-occurring ASD decreased significantly from 13.0 per 1,000 in 1996 to 8.6 per 1,000 in 2010, yet was stable when restricted to 2000 through 2010 (9.5 per 1,000 to 8.6 per 1,000) (Fig 2). The stability in prevalence of ID without ASD from 2000–2010 is consistent with the stable prevalence of ID overall from 1991–2010. The 2010 surveillance year was the first time the overall ASD prevalence surpassed the overall ID prevalence (15.5 and 13.6 per 1,000, respectively), and ASD prevalence without ID was equal to that of ID without ASD (8.6 and 8.6 per 1,000, respectively).

#### Sex, Race/ethnicity, and Level of Intellectual Ability

**Fig 3:**
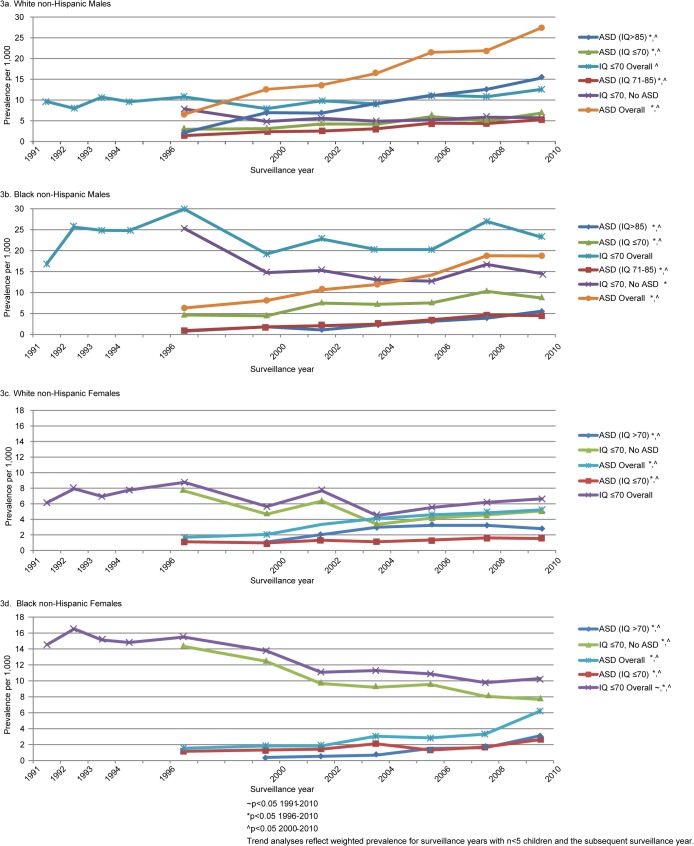
Prevalence of Co-occurring Intellectual Ability and Autism Spectrum Disorder by Sex and Race/ethnicity, 1991–2010.

ASD and Level of Intellectual Ability: Different patterns were observed in the trends for ASD prevalence by sex, race/ethnicity and level of intellectual ability (Fig 3 and Table 2). The prevalence of ASD with average to above average IQ (IQ >85) among NHW males increased from 2.3 per 1,000 in 1996 to 15.3 per 1,000 in 2010, resulting in the highest prevalence estimate across all subgroups by 2010 with an average annual percent increase of 11.3% (8.1, 14.5). While the prevalence of ASD with IQ >85 was significantly higher among NHW than NHB males for each surveillance year, NHB males experienced a greater average annual percent increase of 15.1% (10.7, 19.6).

The prevalence of ASD and borderline IQ (IQ 71–85) in 2010 was similar among NHW and NHB males (5.2 per 1,000 and 4.5 per 1,000, respectively, p = 0.47), yet the average annual percent change was slightly higher among NHB than NHW males [11.6% (9.0, 14.2) and 9.4% (7.8, 11.0), respectively]. The pattern was different among NHW and NHB children with ASD and IQ≤70 (ID). The average annual increase in the prevalence of ASD with ID was greater among NHW males than among NHB males, but the prevalence of ASD with ID was higher among NHB males than NHW males across all surveillance years.

ASD prevalence estimates for NHW and NHB females were lower than those for NHW and NHB males. From 1996 to 2010 and 2000 to 2010, ASD prevalence without ID among both NHB and NHW females increased significantly, most dramatically among NHB females; 24.1% (17.3, 31.2) and 10.4% (4.3, 16.8), respectively. (Table 2 and Fig 3C and 3D). By 2010, ASD prevalence without ID among NHB females surpassed that of NHW females (3.1 per 1,000 and 2.8 per 1,000, respectively). ASD prevalence with co-occurring ID increased significantly for both NHW and NHB females as well. Borderline IQ among females with ASD was not examined due to small sample sizes.

ID without ASD: ID prevalence without ASD among NHW males was stable across both time periods. ID prevalence without ASD among NHB males was stable from 2000–2010 and, in all years, it was approximately three times higher than that observed for NHW males. The stability in prevalence of ID without ASD among both NHW and NHB males from 2000–2010 is consistent with the stable prevalence of ID overall from 1991–2010 for both NHW and NHB males (Fig 3A and 3B). Overall ID prevalence significantly increased from 2000 to 2010 among NHW males.

ID prevalence without co-occurring ASD decreased significantly for NHB females, yet was stable for NHW females. Notably, the prevalence of ID overall among NHB females decreased significantly across all comparison time periods. Similar to that found among males, ID prevalence without ASD was nearly twice as high for NHB compared with NHW females.

### Cerebral palsy

#### Prevalence and demographics

Cerebral palsy (CP) prevalence was stable from 1993 (3.5 per 1,000) through 2010 (3.4 per 1,000) [p<0.33; average annual change of 0.5% (-0.7, 1.6)], with slightly lower estimates in 1991 and 1992 (2.9 per 1,000) resulting in a significant increase in prevalence from 1991–2010, (p<0.05; average annual change of 1.0% (0.01, 1.9)] (Fig 1 and Table 3). Across all surveillance years combined, the prevalence of CP was 3.5 per 1,000 or 1 in 286 children. CP prevalence was higher among NHB than NHW children for all surveillance years except 1993 and 2006. Although a significant increase in prevalence among NHW children was found between 1991 and 2010 [average annual change of 1.4% (0.02, 2.7)], CP prevalence among NHW children was stable from 1993 through 2010 [1.0% (-0.8, 2.8)]. No changes in prevalence were found among NHB or Hispanic children. CP prevalence was consistently higher among males than females, 3.8 and 3.2 per 1,000, respectively across all surveillance years combined.

**Table 3:** Trends in prevalence, demographic characteristics and subtypes of children with cerebral palsy, 1991–2010.

	1991		1992		1993		1994		1996		2000		2002		2004		2006		2008		2010		1991–2010		Average annual percent change 1991–2010~
	%	Prev	%	Prev	%	Prev	%	Prev	%	Prev	%	Prev	%	Prev	%	Prev	%	Prev	%	Prev	%	Prev	%	Prev	
Total	n = 87	2.9	n = 88	2.9	n = 113	3.5	n = 113	3.4	n = 133	3.6	n = 135	3.1	n = 168	3.9	n = 143	3.4	n = 178	4.1	n = 180	4.0	N = 163	3.4	n = 1501	3.5	0.96 (0.01, 1.91)*
**Demographics**																									
White non-Hispanic	54.0	2.8	51.1	2.7	55.8	3.6	43.4	2.8	44.4	3.3	39.3	2.9	36.3	3.6	32.9	2.9	38.2	4.5	32.8	3.9	31.9	3.3	40.2	3.3	1.36 (0.02, 2.72)*
Black non-Hispanic	42.5	3.2	44.3	3.4	38.1	3.4	47.8	4.0	47.4	4.1	51.9	3.6	50.0	4.2	47.6	3.5	43.3	3.8	47.2	4.1	44.2	3.5	46.1	3.7	0.34 (-0.53, 1.21)
Hispanic	nr	nr	3.4	3.1	nr	nr	7.1	6.1	4.5	4.7	3.7	1.5	5.4	2.2	12.6	4.0	11.2	3.8	11.1	3.0	14.1	2.7	7.7	3.0	-0.56 (-4.5, 3.5)
Male	57.5	3.3	47.7	2.7	61.1	4.2	50.4	3.4	52.6	3.8	58.5	3.6	54.2	4.1	57.3	3.8	55.1	4.4	61.7	4.8	50.3	3.3	55.4	3.8	1.08 (-0.23, 2.41)
Female	42.5	2.5	52.3	3.1	38.9	2.8	49.6	3.4	47.4	3.5	41.5	2.6	45.8	3.6	42.7	2.9	44.9	3.8	38.3	3.1	49.7	3.4	44.6	3.2	0.79 (-0.40, 1.99)
**Subtype**																									
Spastic^	74.7	2.2	85.2	2.5	92.0	3.2	86.7	2.9	79.7	2.9	78.5	2.4	73.8	2.9	87.4	3.0	85.4	3.5	75.6	3.0	81.0	2.7	81.5	2.9	0.73 (-0.33, 1.79)
Unilateral	23.1	0.5	26.7	0.7	30.8	1.0	31.6	0.9	44.3	1.3	35.8	0.9	32.3	0.9	32.8	1.0	18.4	0.6	33.8	1.0	27.3	0.7	30.6	0.9	0.14 (-1.95, 2.28)
Bilateral	70.8	1.5	72.0	1.8	67.3	2.2	66.3	1.9	50.9	1.5	61.3	1.5	63.7	1.8	64.0	1.9	75.7	2.7	61.0	1.8	69.7	1.9	65.7	1.9	0.87 (-0.62, 2.38)
Non-spastic**	14.9	0.4	3.4	0.1	6.2	0.2	7.1	0.2	8.3	0.3	12.6	0.4	8.9	0.3	4.2	0.1	7.9	0.3	13.9	0.6	8.0	0.3	8.8	0.3	1.47 (-2.27, 5.36)
CP-NOS^+^	10.3	0.3	11.4	0.3	nr	nr	6.2	0.2	12.0	0.4	8.9	0.3	17.3	0.7	8.4	0.3	6.7	0.3	10.6	0.4	11.0	0.4	9.7	0.3	1.87 (-2.01, 5.91)

* Trend is not significant when restricted to 1993–2010 surveillance

^ Total includes children with spastic-dyskinetic and spastic-ataxic CP, and spastic CP not otherwise specified.

~ Proportions and prevalence estimates with numerators <5 children are suppressed due to small sample sizes. Trend analyses reflect weighted prevalence for surveillance years with n<5 children and the subsequent surveillance year (e.g., trend for Hispanic children with CP combined 1991–1992 numerators and denominators for the first time period).

** Includes ataxic, dyskinetic, hypotonic, and ataxic-dyskinetic CP subtypes.

^+^Includes children with CP not otherwise specified.

#### Subtypes and Co-occurring DDs

The most common subtype was spastic CP, accounting for approximately 82% of CP cases across all surveillance years (range: 74% to 92%). Bilateral spastic CP accounted for most of the spastic CP (51–76%). Among children with CP, approximately 52% also met the case definitions for ID, 15% for VI, 8% for ASD and 4% for HL. Prevalence trends among children with CP and each of the four DDs were stable across the time period.

### Hearing loss

#### Prevalence and demographics

The average annual prevalence of moderate to profound HL from 1991 through 2010 was 1.4 per 1,000 or 1 in 714 children with an average annual change of 0.93% (-0.6, 2.5) (Fig 1 and Table 4). The prevalence of HL was stable within all racial/ethnic and sex subgroups.

**Table 4:** Trends in prevalence, demographic characteristics and severity among children with hearing loss, 1991–2010.

	1991		1992		1993		1994		1996		2000		2002		2004		2006		2008		2010		1991–2010		Average annual percent change 1991–2010
	%	Prev	%	Prev	%	Prev	%	Prev	%	Prev	%	Prev	%	Prev	%	Prev	%	Prev	%	Prev	%	Prev	%	Prev	
Total	n = 39	1.3	n = 41	1.4	n = 29	0.9	n = 49	1.5	n = 52	1.4	n = 53	1.2	n = 64	1.5	n = 49	1.2	n = 59	1.4	n = 83	1.8	N = 64	1.3	n = 582	1.4	0.93 (-0.62, 2.50)
**Demographics**																									
White non-Hispanic	48.7	1.1	36.6	0.9	51.7	0.9	40.8	1.1	53.8	1.5	32.1	0.9	48.4	1.8	24.5	0.7	33.9	1.3	43.4	2.4	35.9	1.4	40.5	1.3	2.79 (-0.18, 5.85)
Black non-Hispanic	43.6	1.5	48.8	1.7	31.0	0.7	53.1	1.9	38.5	1.3	50.9	1.4	37.5	1.2	51.0	1.3	42.4	1.2	28.9	1.2	51.6	1.6	43.0	1.4	-0.41 (-0.25, 1.73)
Male	71.8	1.8	58.5	1.6	44.8	0.8	63.3	1.8	44.2	1.2	58.5	1.4	53.1	1.5	59.2	1.4	42.4	1.1	57.8	2.1	57.8	1.5	55.5	1.5	0.42 (-1.58, 2.47)
Female	28.2	0.7	41.5	1.1	55.2	1.0	36.7	1.1	55.8	1.6	41.5	1.0	46.9	1.4	40.8	1.0	57.6	1.6	42.2	1.6	42.2	1.1	44.5	1.2	1.50 (-0.64, 3.69)
**Level of severity**																									** **
Moderate	38.5	0.5	43.9	0.6	37.9	0.3	46.9	0.7	42.3	0.6	49.1	0.6	43.8	0.6	53.1	0.6	47.5	0.6	55.4	1.0	43.8	0.6	46.6	0.6	1.99 (-0.13, 4.16)
Severe	23.1	0.3	19.5	0.3	27.6	0.2	22.4	0.3	19.2	0.3	17.0	0.2	20.3	0.3	26.5	0.3	23.7	0.3	26.5	0.5	23.4	0.3	22.7	0.3	1.78 (-0.18, 3.79)
Profound	38.5	0.5	36.6	0.5	34.5	0.3	30.6	0.4	36.5	0.5	34.0	0.4	35.9	0.5	20.4	0.2	28.8	0.4	18.1	0.3	32.8	0.4	30.6	0.4	-1.19 (-3.28, 0.94)

#### Severity and Co-occurring DDs

Across all surveillance years, 47% of children with HL had moderate HL, 23% had severe HL, and 31% had profound HL. From 1991 to 2010, the prevalence of HL was stable within each severity level. Sensorineural HL was the predominant type of loss affecting approximately 81% of children with HL. The most common DD to co-occur with HL was ID (23%), followed by CP (10%), ASD (7%), and/or VI (5%). While prevalence estimates for HL with co-occurring CP, VI or ASD were stable from 1991–2010, as previously noted, the prevalence of HL and co-occurring ID significantly increased across this time period.

### Vision impairment

#### Prevalence and demographics

VI prevalence was stable from 1991 (1.0 per 1,000) through 2006 (1.3 per 1,000) with slightly higher estimates in 2008 and 2010 (1.6 and 1.5 per 1,000, respectively); average annual prevalence of 1.3 per 1,000 or 1 in 769 children (Fig 1 and Table 5). The increases in the last two surveillance years resulted in a significant average annual increase in prevalence from 1991–2010 of 1.6% (0.4, 2.9) which was not significant when restricted to 1991 to 2006 [0.7% (-0.9, 2.4)]. A slight, but statistically significant, increase was also found among females [2.1% (0.4, 3.8)] from 1991 to 2010, but not males, NHW or NHB children. VI prevalence among females was stable from 1991–2006; average annual change of 0.9% (-0.9, 2.8).

**Table 5:** Trends in prevalence, demographic characteristics, and severity among children with vision impairment, 1991–2010.

	1991		1992		1993		1994		1996		2000		2002		2004		2006		2008		2010		1991–2010		Average annual percent change 1991–2010
	%	Prev	%	Prev	%	Prev	%	Prev	%	Prev	%	Prev	%	Prev	%	Prev	%	Prev	%	Prev	%	Prev	%	Prev	
Total	n = 29	1.0	n = 41	1.4	n = 34	1.1	n = 36	1.1	n = 50	1.4	n = 48	1.1	n = 45	1.0	n = 57	1.3	n = 55	1.3	n = 73	1.6	N = 71	1.5	n = 539	1.3	**1.60 (0.35, 2.86)**
**Demographics**																									
White non-Hispanic	41.4	0.7	48.8	1.2	64.7	1.2	58.3	1.2	50.0	1.4	31.3	0.8	44.4	1.2	24.6	0.9	32.7	1.2	27.4	1.3	28.2	1.3	38.4	1.1	0.55 (-1.41, 2.55)
Black non-Hispanic	51.7	1.3	46.3	1.6	32.4	0.9	36.1	1.0	42.0	1.4	58.3	1.4	42.2	1.0	54.4	1.6	40.0	1.1	38.4	1.4	36.6	1.3	43.2	1.3	0.22 (-1.73, 2.21)
Male	44.8	0.9	61.0	1.6	61.8	1.3	52.8	1.1	58.0	1.6	62.5	1.4	46.7	1.0	52.6	1.4	58.2	1.5	47.9	1.5	56.3	1.6	54.7	1.4	1.21 (-0.53, 2.98)
Female	55.2	1.1	39.0	1.1	38.2	0.8	47.2	1.0	42.0	1.2	37.5	0.8	53.3	1.1	47.4	1.3	41.8	1.1	52.1	1.7	43.7	1.3	45.3	1.2	**2.06 (0.39, 3.77)**
**Level of severity**																									
Low vision	31.0	0.3	31.7	0.4	11.8	0.1	16.7	0.2	28.0	0.4	31.3	0.3	28.9	0.3	26.3	0.4	30.9	0.4	31.5	0.5	33.8	0.5	28.4	0.4	**3.37 (0.54, 6.28)**
Blindness	69.0	0.7	68.3	0.9	88.2	0.9	75.0	0.8	58.0	0.8	64.6	0.7	71.1	0.7	73.7	1.0	69.1	0.9	64.4	1.0	57.7	0.8	67.7	0.9	0.77 (-0.48, 2.05)
VI NOS*	nr	nr	nr	nr	nr	nr	nr	nr	14.0	0.2	nr	nr	nr	nr	nr	nr	nr	nr	nr	nr	8.5	0.1	3.9	0.05	N/A

*Proportions and prevalence estimates with numerators <5 children are suppressed due to small sample sizes.

#### Severity and Co-occurring DDs

Across all surveillance years, the majority (68%) of children with VI met the definition for legal blindness with 28% having low vision. An increase in prevalence was found among children with low vision [3.4% (0.5, 6.3)]. Of children with VI, 55% had co-occurring ID followed by 42% with CP, 8% with ASD and 5% with co-occurring HL. Prevalence estimates of VI and co-occurring ID, CP, HL and/or ASD were stable from 1991 through 2010.

## Discussion

As school age children with DDs grow into adolescence and transition into adulthood, appropriate planning for services and resources is critical. MADDSP’s ongoing collection of prevalence data provides insight into the changing characteristics of children with DDs and provides a more thorough understanding of their present and future support needs. In addition, assessment of the co-occurrence of DDs may suggest areas for further investigation regarding shared risk factors and etiologies.

Improved neonatal survivorship, particularly among infants born low birth weight and preterm, has raised concern that the prevalence of DDs associated with these birth characteristics would concurrently increase [26]. Of the five DDs monitored by MADDSP, prevalence estimates for ID, CP, HL, and VI were relatively stable over the 20-year period with remarkable increases in ASD prevalence over its 15-year monitoring period. As no declines in prevalence were observed, our findings underscore the persistent and significant community resources needed to serve children with ID, CP, HL, VI, and ASD.

The stability of overall ID, CP, HL and VI prevalence might reflect a balance between increased potential for disability as more infants survive the neonatal period and reductions in other risk factors. For example, hearing loss is associated with prematurity and thus, increased survival of preterm infants might increase HL prevalence. However, HL is also a common sequela of bacterial meningitis which has declined due to vaccines that became available during the study period such as that for Haemophilus influenzae type B [27–29]. Stable prevalence may also reflect opposing trends across DD severity levels or specific birth characteristics. Data from the Surveillance of CP in Europe (SCPE) collaboration reported decreases in birth prevalence of spastic bilateral CP among children born NBW accompanied by increases in spastic unilateral CP among infants of comparable birth weight [30]. MADDSP’s slight increases in VI prevalence in 2008 and 2010 as well as higher prevalence estimates of co-occurring ID and HL in 2006 and 2008 could indicate greater exposure to unidentified risk factors in later surveillance years and may support potential changes in the characteristics and etiology of children with these DDs in metropolitan Atlanta. Of interest and encouragement, was the significant decline in ID prevalence among NHB females across all time period. Analyses examining the influences of differential testing practices, migration, and perinatal risk factors on DD prevalence trends among various subgroups are necessary and underway to better understand this and other MADDSP findings.

In contrast to the other DDs, ASD prevalence increased 269% from 1996 to 2010, with an estimated average annual increase of 9.3%, a finding consistent with previous studies. Studies based on US survey data demonstrated an approximate 290% increase in parent-reported ASD prevalence between 1997–1999 and 2006–2008 [6] and a 67% increase in prevalence between 2007 *and* 2011–2012 [31]. Aggregate eligibility data from the US Office of Special Education Programs and administrative diagnostic data from the California Department of Developmental Services have demonstrated consistent increasing trends in ASD, as well [8–9]. Previously published data from the ADDM Network showed a 78% increase from 2002 to 2008 [32] and a 123% increase from 2002 to 2010 [5].

Whether and to what extent the identified ASD trend reflects improved identification or changes in the number and/or severity of population risk factors is of keen interest [33]. The contributions of these two possible explanations are complex, difficult to measure, and not easily separable given currently available data [4]. A recent statistical modelling analysis assessed a range of perinatal risk factors including prematurity, low birth weight, multiple birth, cesarean delivery, breech presentation, and assisted reproductive technology, for ASD and demonstrated that none had sufficient characteristics—baseline prevalence, change in prevalence over time, and magnitude of association with ASD—to have substantially impacted identified ASD prevalence over time [34]. More research on other risk factors influencing the ASD prevalence increase is needed.

With respect to identification, there has been considerable discussion over the degree to which changes in diagnostic standards and practices contribute to increases in ASD prevalence, particularly in relation to changes or stability in the prevalence of ID [8–10]. Since MADDSP obtains IQ data to estimate ID prevalence rather than rely on a diagnosis of ID, comparison of trends in ID and ASD prevalence overall and their co-occurrences reflect the accretion of an ASD diagnosis or documentation of behaviors consistent with ASD among children with an IQ ≤70 [10]. It is clear that over time the prevalence of ASD has increased dramatically among children with and without an IQ ≤70 across all sex and racial/ethnic groups. This is concurrent with stability in ID prevalence overall from 1991 to 2010 and 2000 to 2010, a significant decrease in ID prevalence among NHB females across all time period comparisons, and a significant increase in ID prevalence among NHW males from 2000 to 2010. The occurrence of diagnostic substitution (i.e., i.e., switch from one diagnosis to another) may influence ID prevalence, as measured by IQ data, if increased awareness of ASD resulted in changes in administration of psychometric testing. MADDSP data showed a decrease in ID prevalence among NHB females concurrent with an increase in ASD prevalence suggesting the potential for diagnostic substitution contributing to the rise in ASD prevalence among this subgroup only. It is unclear why changes in testing practices would only impact NHB females and NHW males. Examination of the types and timing of evaluations and standardized tests is underway for all sex and racial/ethnic subgroups.

Given that level of intellectual ability has been reported as an important predictor of functioning for children with ASD, the disparity we observed in intellectual ability by race/ethnicity is concerning. In 2010, approximately 75% of NHW males with ASD had IQ >70 compared with only 54% of NHB males with a similar disparity in proportions observed between NHW and NHB females. This racial disparity could be attributed to under-ascertainment of ASD among NHB children without intellectual disability [35], factors related to socioeconomic status {e.g., inadequate cognitive stimulation [36] or environmental lead exposure [37–38], disparities in access to diagnostic evaluations for children with ID or other severe DDs, existing racial/ethnic disparities in ID prevalence, and/or racial differences in cognitive testing practices (e.g., differential use of nonverbal tests) [39]. If the hypothesis of under-ascertainment of ASD among NHB boys without ID is correct, our data showing the greatest annual percent increases in ASD prevalence among NHB children without ID could predict a reduction of this racial/ethnic disparity in future surveillance years. ASD prevalence among NHB children may increase further with increased awareness among community providers and universal screening programs as recommended by the American Academy of Pediatrics [40]. This could subsequently improve documentation of diagnoses and/or behaviors consistent with MADDSP’s ASD surveillance case definition. Further exploration is needed to understand disparities in the timing of evaluation and diagnosis of ASD as well as potential factors, including socioeconomic status, influencing differences across subgroups in access to services.

### Strengths

This study adds to the very limited literature on trends in population-based prevalence of DDs in the U.S. and provides in-depth assessments of ASD trends by level of intellectual ability within demographic subgroups along with comparable assessments and co-occurrence of four other DDs – ID, CP, HL, and VI. Collection of all behavioral descriptions, physical findings, and objective test data from multiple qualifying education and health records to determine case status affords the ability for identification of surveillance cases beyond those identified by community providers. Furthermore, the consistent application of methods and case definitions across all surveillance years provides strong internal validity to evaluate trends. Ascertainment of ID based on standardized IQ measurements is a particular strength of the MADDSP approach. Although adaptive functioning data are not included in the MADDSP ID case definition, analyses indicated that its inclusion would have lowered ID prevalence due to missing data rather than exclusion of children with both an IQ and adaptive functioning test score of ≤ 70 [41]. As Atlanta has a heterogeneous population, we were able to examine prevalence trends for DDs within various race/ethnicity subgroups.

### Limitations

MADDSP relies on both the availability and quality of information in source records to estimate and describe ASD prevalence. Some records for children eligible for screening could not be located. In previous evaluations we found that the effect of missing records likely resulted in a minimal underestimation of ASD prevalence, ranging from 1.1% to 4.3% for the 5 surveillance years for which this could be evaluated [5, 32, 42–44]. Similarly, the inability to access education records for children enrolled in private schools, enrolled in public schools but not receiving special education services, or being homeschooled may contribute to under-ascertainment. Information from the Atlanta Area Association of Independent Schools (AAAIS) indicates that few private schools in metro Atlanta serve children with severe DDs [45]. Since families of children with ASD using specialized private education services might also be more likely to have sought clinical diagnostic services in the Atlanta area, many of which are participating MADDSP providers, the likelihood that private and homeschooled children would be missed by MADDSP may be low. An assessment of the validity of MADDSP ASD methods against the gold standard of an in-person evaluation found MADDSP ASD estimates to be conservative with fairly high positive predictive value [79%, (95% CI 66%-9.3%)], but lower sensitivity [60%, (95%CI 45%-75%)], consistent with exclusion of children who are not receiving special education services in public school and are not served by MADDSP clinical sources [46].

While challenging to quantify, a better understanding is needed of how the quality of information in available records may be improving over time and thus affecting ASD estimates. As case definitions for ID, CP, HL, and VI are based on objective test and physical finding information, these estimates are likely not susceptible to changes over time in the quality of information in records. In addition, IQ scores were not available in source records for all children. This may have resulted in an underestimate of co-occurring ID among children with CP, VI, and HL, as children for whom IQ information was unavailable, due to either a child being untestable or to a formal IQ test not being given, were not excluded from the denominator.

The cross-sectional nature of MADDSP data did not allow us to evaluate our case children longitudinally to determine whether their diagnostic or service classifications changed over time. Furthermore, as ASD surveillance was added to MADDSP in 1996, significantly more resources were needed to monitor all five DDs. As a result the next surveillance year was 2000 and, therefore, it is not possible to estimate ASD and ID prevalence in 1998 or additional intervening years to further understand the high ID prevalence in 1996.

## Conclusion

Since DDs frequently occur together, evaluation of concurrent trends for related disabilities in the same population, using the same methodology provides the opportunity to assess changes in community identification and classification as well as suggest hypotheses regarding potential etiology. Information on intellectual ability improves our understanding of differences in community identification of ASD, particularly within specific subgroups. The stability of ID, CP, HL and VI prevalence, despite improvements in neonatal survival, is encouraging, yet decreases in prevalence of these DDs were not found. These results support the need to continue to monitor trends as well as to accelerate the pace of etiologic and risk factor research to identify areas for prevention. The complexity of evaluating and supporting children with ASD and their families coupled with persistent growth in prevalence highlights the significant resource challenges faced by communities [47]. Comparing trends in shared risk profiles among children with DDs with those of their counterparts without DDs may guide future epidemiologic research, service planning and provision efforts. Most importantly, these data emphasize the significant and ongoing community resources required to support children with DDs and their families.

## Supporting Information

S1 Appendix Population denominators for 8-year-olds in Metropolitan Atlanta, 1991–2010. (DOCX)

S2 Appendix Terminology for population estimates. (DOCX)
