# Supplementary material for: Trends in the Prevalence of Autism Spectrum Disorder, Cerebral Palsy, Hearing Loss, Intellectual Disability, and Vision Impairment, Metropolitan Atlanta, 1991–2010
Source: PLoS One. 2015 Apr 29;10(4):e0124120. doi: 10.1371/journal.pone.0124120 (PMC4414511; doi:10.1371/journal.pone.0124120)
Supplement: S2 Appendix — (DOCX) [file pone.0124120.s002.docx]

Bridged-race: Race bridging is a method used to make multiple-race and single-race data collection systems sufficiently comparable for estimation and analysis of race-specific statistics at a point in time or over time. These estimates result from bridging the 31 race categories used in United States (US) Census 2000 and US Census 2010, as specified in the 1997 Office of Management and Budget (OMB) standards for the collection of data on race and ethnicity, to the four race categories specified in the 1977 OMB standards (<http://www.cdc.gov/nchs/nvss/bridged_race.htm> Accessed January 27, 2015).

Decennial population estimates: A complete enumeration of the US population taken by the US Census Bureau in each year ending in zero (<http://www.census.gov/dmd/www/glossary.html#I> Accessed January 27, 2015).

Postcensal population estimates: Population estimates produced for the years after a decennial census when only the beginning population is known. They are produced and revised each year. For dates when both postcensal and intercensal estimates are available, intercensal estimates are preferred ( <http://www.census.gov/popest/about/terms.html> Accessed January 27, 2015).

Intercensal population estimates: Population estimates produced for the years between two decennial censuses when both the beginning and ending populations are known. They are produced once a decade by adjusting the existing time series of postcensal estimates for the entire decade to smooth the transition from one decennial census count to the next. They differ from the postcensal estimates that are released annually because they rely on a mathematical formula that redistributes the difference between the April 1 postcensal estimate and April 1 census count for the end of the decade across the estimates for that decade. For dates when both postcensal and intercensal estimates are available, intercensal estimates are preferred.( <http://www.census.gov/popest/about/terms.html> Accessed January 27, 2015).
